# Normalizing salt content by mixing native human airway mucus samples normalizes sample rheology

**DOI:** 10.3389/fphys.2023.1111647

**Published:** 2023-03-10

**Authors:** Matthew R. Markovetz, Jacob E. Hibbard, Lucas M. Plott, Lawrence G. Bacudio, William J. Kissner, Andrew Ghio, Priya A. Kumar, Harendra Arora, David B. Hill

**Affiliations:** ^1^ Marsico Lung Institute, The University of North Carolina at Chapel Hill, Chapel Hill, NC, United States; ^2^ National Health and Environmental Effects Research Laboratory, U.S. Environmental Protection Agency, Chapel Hill, NC, United States; ^3^ Department of Anesthesiology, The University of North Carolina at Chapel Hill, Chapel Hill, NC, United States; ^4^ Outcomes Research Consortium, Cleveland, OH, United States; ^5^ Joint Department of Biomedical Engineering, North Carolina State University and the University of North Carolina at Chapel Hill, Chapel Hill, NC, United States

**Keywords:** mucus, mucus rheology, particle tracking microrheology, airway physiology, muco-obstructive pulmonary diseases

## Abstract

Across the globe, millions of people are affected by muco-obstructive pulmonary diseases like cystic fibrosis, asthma, and chronic obstructive pulmonary disease. In MOPDs, the airway mucus becomes hyperconcentrated, increasing viscoelasticity and impairing mucus clearance. Research focused on treatment of MOPDs requires relevant sources of airway mucus both as a control sample type and as a basis for manipulation to study the effects of additional hyperconcentration, inflammatory milieu, and biofilm growth on the biochemical and biophysical properties of mucus. Endotracheal tube mucus has been identified as a prospective source of native airway mucus given its several advantages over sputum and airway cell culture mucus such as ease of access and *in vivo* production that includes surface airway and submucosal gland secretions. Still, many ETT samples suffer from altered tonicity and composition from either dehydration, salivary dilution, or other contamination. Herein, the biochemical compositions of ETT mucus from healthy human subjects were determined. Samples were characterized in terms of tonicity, pooled, and restored to normal tonicity. Salt-normalized ETT mucus exhibited similar concentration-dependent rheologic properties as originally isotonic mucus. This rheology agreed across spatial scales and with previous reports of the biophysics of ETT mucus. This work affirms previous reports of the importance of salt concentration on mucus rheology and presents methodology to increase yield native airway mucus samples for laboratory use and manipulation.

## 1 Introduction

The human airway surface liquid is composed of two layers that clear inhaled particles and pathogens from the lung thus protecting its (Button et al., 2012). The mucus layer is a gel network with structure primarily composed of mucins MUC5B and, to a lesser extent, MUC5AC (Okuda et al., 2019; Radicioni et al., 2021). The periciliary layer is composed of cilia and surface-bound mucins that serve to both lubricate beating cilia and as a size exclusion barrier. Ciliary beat drives mucus flow from the distal to proximal airways resulting in mucociliary clearance, a primary defense mechanism of the airways (Hill et al., 2022). Recent work has shown that of many parameters that affect mucus biophysics, including pH and salt concentration, the concentration of mucus itself is the primary driver of its rheological behavior (Hill et al., 2014; Hill et al., 2018; Markovetz et al., 2019). In particular, mucus hyperconcentration is the chief physiological derangement responsible for the pathophysiology of muco-obstructive pulmonary diseases like asthma, cystic fibrosis, and chronic obstructive pulmonary disease that affect millions of patients worldwide (Boucher, 2019; Hill et al., 2022). Mucus concentration also determines a number of the complex, less-understood behaviors of mucus as they relate to osmotic pressure and overlap-entanglement phase behaviors (Hill et al., 2022).

Because the need for useful models of mucus is so great, a number of airway mucus analogs have been recently developed. These range from apically secreted mucus harvested from human bronchial epithelial cell cultures to synthetic mucus analogs reliant on lab-isolated mucins like porcine gastric mucin or even synthetic polymers to recapitulate the viscoelastic behavior of airway mucus (Hill et al., 2014; Huck et al., 2019). Recently, however, efforts have been made to generate representative “stocks” of native human airway mucus for laboratory use to study the biophysics of mucus and its response to therapeutic and environmental challenges. One such approach has been to recover mucus from the tips of endotracheal tubes (ETT) used to ventilate patients during outpatient surgeries (Markovetz et al., 2019). These samples contain proteins secreted onto distal and proximal conducting airways, as well as submucosal glands (Kato et al., 2022), making them highly representative in terms of biological composition. However, due to evaporative loss of water during transport or preparation or washing to clear partial obstruction, these samples tend to be non-isotonic. Because tonicity also effects biophysical and biochemical properties in mucus the salt concentration must be controlled for repeatable experimentation (Wagner et al., 2017). A straightforward way to do so is to use only ETT samples with isotonic salt concentrations. However, such samples appear to make up only ∼20% of all samples recovered (Markovetz et al., 2019), which represents a target for improvement given the effort and resources taken to characterize and identify high quality samples for laboratory use.

In this study, we sought to determine whether non-isotonic ETT samples could be pooled in a similar manner to isotonic samples. To do so, we sought to achieve an isotonic mixture from a combination of either hypo- or hypertonic samples (or both). We do so principally to demonstrate the utility of non-isotonic samples and improve collection yield. Isotonic and non-isotonic samples collected during a one-week period were characterized and pooled and their biophysical behaviors compared at the microscopic and bulk scales. Hypotonic samples were also pooled and brought to isotonicity through salt addition. We studied these pools at original and diluted concentrations to probe behaviors near the purported entanglement concentration of mucus (∼2% solids) (Georgiades et al., 2014). We conclude that non-isotonic samples are equally appropriate for laboratory use as isotonic samples when pooled and mixed to achieve physiological tonicity, and that ETT mucus follows a uniform scaling behavior in line with what has been suggested previously.

## 2 Materials and methods

### 2.1 Endotracheal tube collection and mucus recovery

ETTs were collected at the University of North Carolina (UNC) Hospital in Chapel Hill as approved by the UNC IRB (#11-0413). The tips (approximately the last 10 cm) of the ETTs were removed upon completion of surgical procedures. The tips were placed in a 50 mL conical tube, set on ice for transport to the UNC Marsico Lung Institute, and centrifuged at 400 g upon delivery to harvest the mucus. Each tip yielded hundreds of microliters of mucus (see Tables 1, 2) in line with previous reports. Samples were snap-frozen and stored at −80°C to reduce proteolysis. Samples with blood contamination, odd coloration, or obtained from subjects with acute or chronic lung disease were excluded from this study.

**TABLE 1:** Isotonic (ISO) Samples—5 ETT samples collected the week of 01/22/2020-01/29/2020 were found to have physiological Na^+^ and K^+^ concentrations, fitting the characterization of “isotonic” as previously published. Fold concentration and fold concentration with K specify the proportional difference in tonicity of sample using either Na^+^ concentration alone or with K^+^. The 2(Na + K) metric is a surrogate measure for total electrolyte concentration.

Age	Sex	Race	Sample Vol (*μ1*)	% solids	Na-F(mM)	K^-^F (mM)	Na/K Ratio	Fold Conc.	Fold Conc. w/*K*	2(Na + N) (mM)
29	M	Black	75	4.76	119.09	20.47	5.82	0.99	1.18	279.12
51	F	Unknown	300	5.38	121.77	15.24	7.99	1.01	1.02	274.01
18	M	Hispanic	275	3.25	110.44	7.89	14.00	0.92	0.72	236.65
75	F	Caucasian	700	7.44	102.64	16.23	6.33	0.86	0.97	237.73
49	M	Black	800	3.98	108.71	13.57	8.01	0.91	0.91	244.57
Mean			430	5.01	112.53	14.68	8.43	0.94	0.96	254.42

**TABLE 2:** Non-isotonic (MIX) Samples—3 ETT samples collected the week of 01/22/2020-01/29/2020 were found to have a physiological Na^+^ and K^+^ concentrations but had a pooled average cation concentration satisfying isotonic constraints.

Age	Sex	Race	Sample vol *(μ1)*	% solids	Na+ (mM)	K+ (mM)	Na/K Ratio	Fold Conc.	Fold conc. w/*K*	2(Na + k) (mM)
62	M	Black	2000	7.14	99.67	13.14	7.58	0.83	0.85	225.64
61	F	Cauc	330	4.49	97.14	9.27	10.48	0.81	0.71	212.82
64	M	Cauc	250	5.15	155.49	15.88	9.79	1.30	1.18	342.73
Mean			860	5.60	117.4	12.76	9.28	0.98	0.91	260.40

### 2.2 Measurement of mucus Na^+^ and K^+^ concentrations

Using similar methods to those described in previous studies (Markovetz et al., 2019), 10 µL aliquots of each ETT mucus were used for determination of Na^+^ and K^+^ concentrations. Mucus samples were diluted into an equal volume of 6 N HCl/10% trichloroacetic acid and maintained at 70°C for 24 h (Optima, Fisher, Pittsburgh, PA). Following centrifugation, Na^+^ and K^+^ concentrations were measured in the supernatant *via* inductively-coupled plasma optical emission spectrometry (ICP-OES) at wavelengths of 589.592 and 766.490 nm respectively (Optima 4300 DV; PerkinElmer, Norwalk, CT). A multi-element standard (VHG Labs, Manchester, NH) was used for the calibration curve. Quality assurance checks were obtained using a second multi-element standard (SPEX CertiPrep, Metuchen, NJ).

### 2.3 Pooling and dilution of mucus for rheological characterization across concentrations

“Stocks” of isotonic (ISO, Table 1) and non-isotonic (MIX, Table 2) ETT mucus were generated by pooling 250 µL aliquots from each ETT sample described in each respective table. A salt-corrected pool (ADD, Table 3) was generated from visually acceptable samples (i.e., light colored with minimal-to-no blood contamination) that we found to be ∼50% normal tonicity on average. NaCl and KCl were added to a pool of 100 µL of each sample in the ADD group to normalize tonicity of the pooled stock, and pool isotonicity was verified *via* ICP-OES. The samples were thawed, combined, and mixed *via* trituration in the presence of protease inhibitors (Roche Diagnostics, Germany) to prevent sample degradation. Each stock was then mixed overnight on a rotator at 4°C, as previously described (Markovetz et al., 2019).

**TABLE 3:** Tonicity corrected samples by way of salt addition (ADD)—9 ETT samples were found to have similarly sub-physiological Na^+^ and K^+^ concentrations (∼50% of normal).

Age	Sac	Race	Sample Vol (*μ*1)	% solids	Na+ (mM)	K+ (mM)	Na/K Ratio	Fold Conc.	Fold conc. w/*K*	2(Na + A) (mM)
64	F	Caucasian	500	4.51	78.64	6.03	13.0	0.53	0.66	169.34
73	F	Caucasian	300	3.57	62.14	16.58	3.8	0.81	0.52	157.44
40	F	Caucasian	325	3.77	65.55	4.47	14.7	0.42	0.55	140.04
66	M	Caucasian	300	5.22	76.67	23.62	3.25	1.11	0.64	200.58
41	F	Black	500	4.46	89.04	14.99	5.94	0.87	0.74	208.06
76	M	Caucasian	400	9.69	51.85	16.2	3.20	0.76	0.43	136.1
67	F	Caucasian	300	5.47	68.93	9.59	7.19	0.61	0.57	157.04
59	F	Caucasian	300	2.03	60.9	7.07	8.61	0.49	0.51	135.94
72	M	Caucasian	250	4.55	40.62	11.43	3.55	0.55	0.34	104.1
Mean			353	4.80	66.04	12.22	7.02	0.68	0.55	156.5156

### 2.4 Rheological characterization of ETT mucus stocks

Characterization of the bulk viscoelasticity of ETT mucus at designated concentrations was performed *via* cone (20 mm diameter, 1° deflection) and plate rheometry. Forty microliters of mucus was loaded onto the Peltier plate of a DHR-3 rheometer (TA Instruments, New Castle, DE). Oscillatory shear testing was performed over a range of small strain magnitudes (0.1%-10% strain) to determine the linear viscoelastic regime (LVR) for the storage (G′) and loss (G”) moduli. In the LVR, G′ and G″ are independent of shear strain, allowing for the frequency dependence of the viscoelastic moduli to be assessed across a range of 0.1-10 radians/s^6^. This methodology was used for all mucus types studied with at least 3 technical replicates per sample type. Cone and plate rheological measurements of G’ and G″ that characterize the macroscopic properties of the mucus gel were complemented with particle tracking microrheology (PTMR). PTMR assesses the mechanical properties of mucus at the length-scale of its constituent biopolymers (Lai et al., 2009; Schuster et al., 2013). Briefly, the thermally driven motion of 1 µm diameter carboxylated Fluospheres (ThermoFisher, Fremont, CA), henceforth referred to as “beads”, was tracked to determine the viscoelastic properties of the gel. Bead motion was recorded for 30 s at a rate of 60 frames/s using a ×40 air objective on a Nikon Eclipse TE 2000U microscope. Individual bead trajectories were measured automatically using a custom Python program that uses TrackPy (doi: 10.5281/zenodo.34028) for bead localization and tracking. Bead motion was converted into mean squared displacements (MSD) and complex viscosity (η*) values in accordance with the mathematics described previously (Mason, 2000; Hill et al., 2014; Markovetz et al., 2019).

### 2.5 Statistical methods

All statistical analyses were performed in Matlab (© 2022The MathWorks, Natick, MA). Differences in measured values or trends were deemed statistically significant where *p* < α = 0.05. Differences between sample means were assessed *via* Wilcoxon Rank Sum Test using the *ranksum* function in Matlab, and differences in trends were assessed using the Kruskal-Wallis test with the Matlab function *kruskalwallis*.

## 3 Results

### 3.1 Sample description and selection based on ionic content

Mucus samples from 18 adult subjects undergoing elective surgery at the University of North Carolina hospital were collected during a one week period (01/22/2020–01/29/2020). From this collection period, 8 samples met our inclusion criteria and were separated into an *n* = 5 “isotonic” (ISO, Table 1) and *n* = 3 “non-isotonic” (MIX, Table 2) group for pooling. Reasons for sample exclusion were visible blood contamination (*n* = 3) and COPD diagnosis (*n* = 2). The other 5 unused samples had low volumes (<100 µL) or tonicities (Na + K < 70 mM) that would have yielded a less optimal mixture (i.e., further from normal tonicity) than those combined to form the MIX pool. An additional (ADD, Table 3) pool composed of *n* = 9 similar hypotonic samples procured during weekly collections from January 2020 through May 2022 was generated (following a one year-long pause due to the COVID-19 pandemic). ADD samples met all sample quality criteria for selection and were brought to normal tonicity during pooling. There were no sex-dependent effects on sample tonicity.

### 3.2 Sample volume and solids content

**FIGURE 1:**
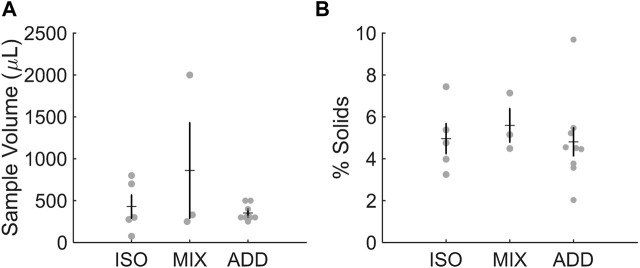
Characteristics of individual ETT samples for each pool studied. **(A)** Sample volume and **(B)** % solids were in range of previous reports.

We previously reported that collected volumes of ETT mucus samples averaged 581 µL (±228 µL) (Markovetz et al., 2019). The average volume of samples collected in the current study approximates this range (Figure 1A). ISO samples reported average volumes of 430 μL at the time of procurement, while the MIX group averaged 860 µL due to an abnormally large sample volume (2 mL) recovered from one patient. ADD samples averaged 353 µL prior to tonicity normalization. Despite some variance in sample volume across groups, % solids were similar on average for ISO (5.0%), MIX (5.6%), and ADD (4.8%), which is similar to previous reports of ∼5% solids from recovered ETT mucus samples (Figure 1B). There were no sex-dependent effects on sample volume or concentration.

### 3.3 Macrorheological properties of mucus

**FIGURE 2:**
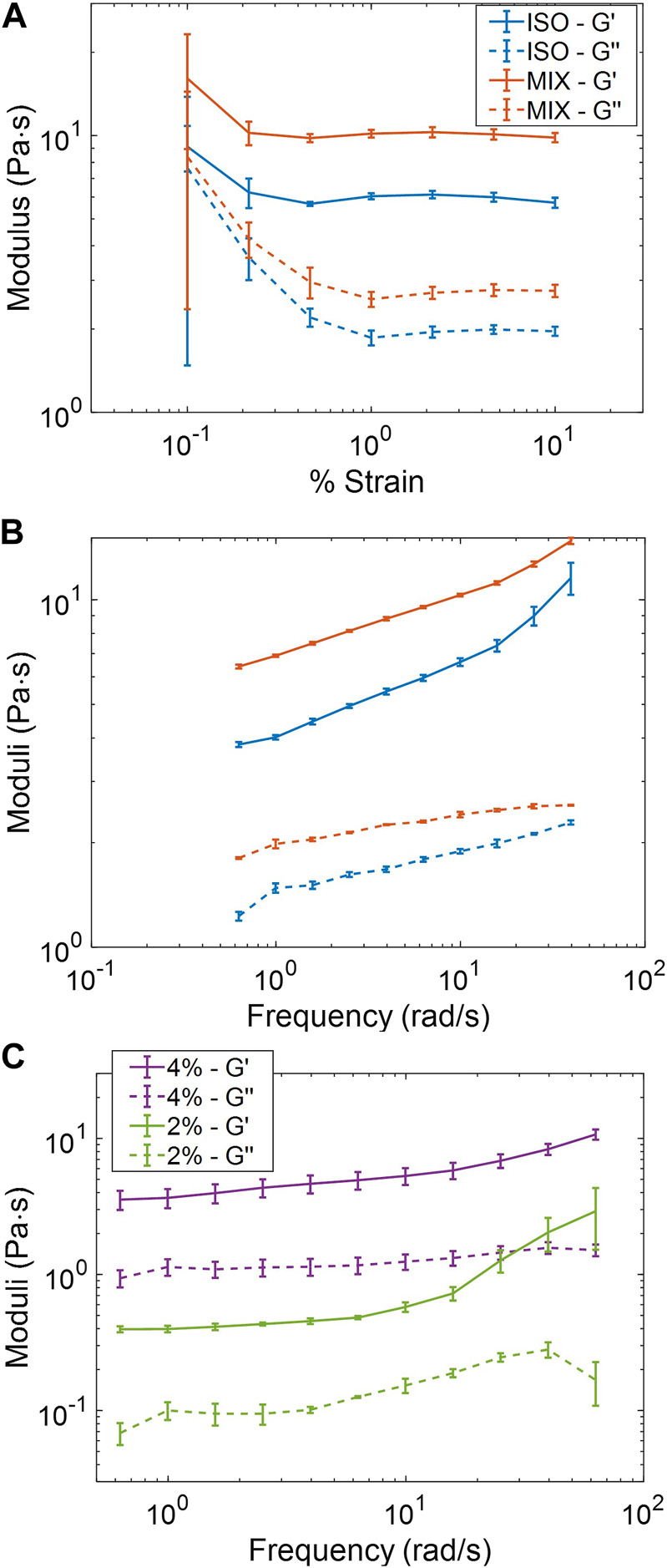
Macrorheological behavior of pooled ETT mucus samples. **(A)** ISO (5%) and MIX (5.6%) pools collected the same week demonstrated qualitatively similar behavior and an LVR at ≥ 1% strain. **(B)** ISO and Mix pools displayed qualitatively similar frequency responses with clear gel-like behavior. **(C)** Frequency response of the ADD pool at two concentrations.

The viscoelastic properties of ISO and MIX pools from the same week of sample collection were compared directly *via* cone-and-plate rheometry. In a strain-sweep assay to determine the linear viscoelastic regime of each pooled sample, the ISO pool had decreased storage (G′) and loss (G″) moduli compared to the more concentrated MIX pool (Figure 2A), though both samples had similar qualitative responses across the range of strains explored (0.1%–10%). As in previous studies, we examined the frequency response of the samples at 1% strain, and the MIX pool demonstrated increased G′ and G″ relative to the ISO group due to differences in sample concentration (Figure 2B). However, both samples behaved in a qualitatively similar manner with tan(δ) < 0.4 across the range of frequencies examined, indicating both samples behaved as hydrogels. The ADD pool was brought to isotonicity and diluted in PBS to 4% and then 2% solids to assess the tunability of initially non-isotonic samples *via* a frequency sweep at 1% strain. As expected, the 4% pool reported moduli roughly ten-fold in excess of the 2% pool (Figure 2C) with tan(δ) ≈ 0.3 across the frequency spectrum for both samples. These results indicate that salt-normalized ETT mucus behaves predictably as a hydrogel and is tunable to reflect the concentration-dependent nature of airway mucus physiology and pathophysiology.

### 3.4 Particle tracking microrheology (PTMR)

**FIGURE 3:**
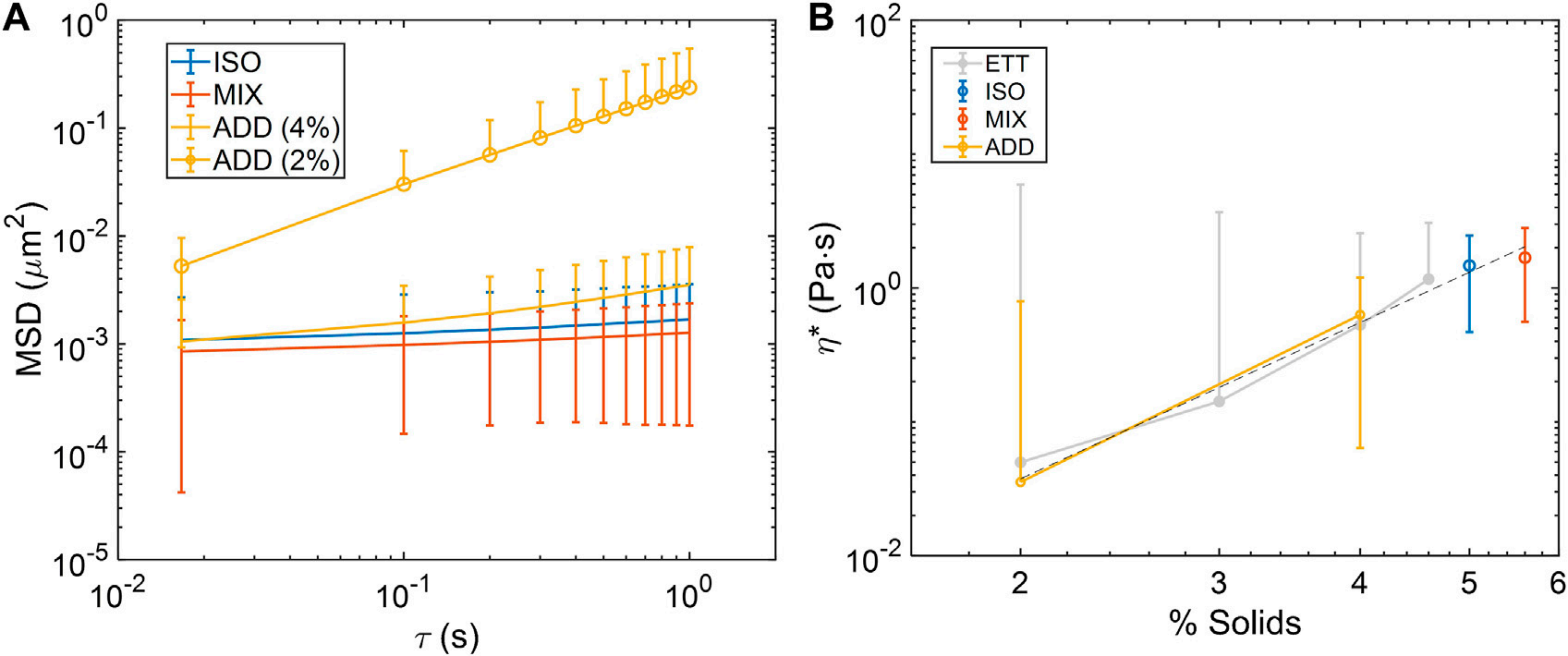
Microrheological characterization of ETT mucus pools. **(A)** Mean squared-displacement (MSD) curves of bead diffusion in pooled mucus samples at various concentrations. **(B)** Complex viscosity (η*) calculated from MSD in each pool at the examined concentrations follows the power law scaling behavior of η*∼c^3.9^ (dashed line), which is nearly identical to η*∼c^3.8^ in previously reported isotonic ETT data (light grey) (Markovetz et al., 2019). Data obtained from *n* = 3 technical replicates.

Thermal motion of 1 μm carboxylated polystyrene beads was tracked, and mean-squared displacement (MSD) values were calculated for all lag-times in each 1,200 frame (60 fps) video. As with bulk cone-and-plate rheometry, rheological behavior in MIX, ISO, and both ADD pools was concentration-dependent, with MSD increasing as concentration decreased (Figure 3A). MSD was converted into complex viscosity (η*) *via* the general Stokes-Einstein equation, and viscosity-concentration relationship of η* ∼ c^3.9^ was evident, which is nearly identical to previous reports in ETT mucus other mucus types (Figure 3B) (Georgiades et al., 2014; Markovetz et al., 2019). These results indicate that ETT mucus, when salt-normalized, follows a repeatably uniform rheological scaling law at the micro-scale in mucus ranging from normal to pathophysiological concentrations.

### 3.5 Macro and microrheological comparison

**FIGURE 4:**
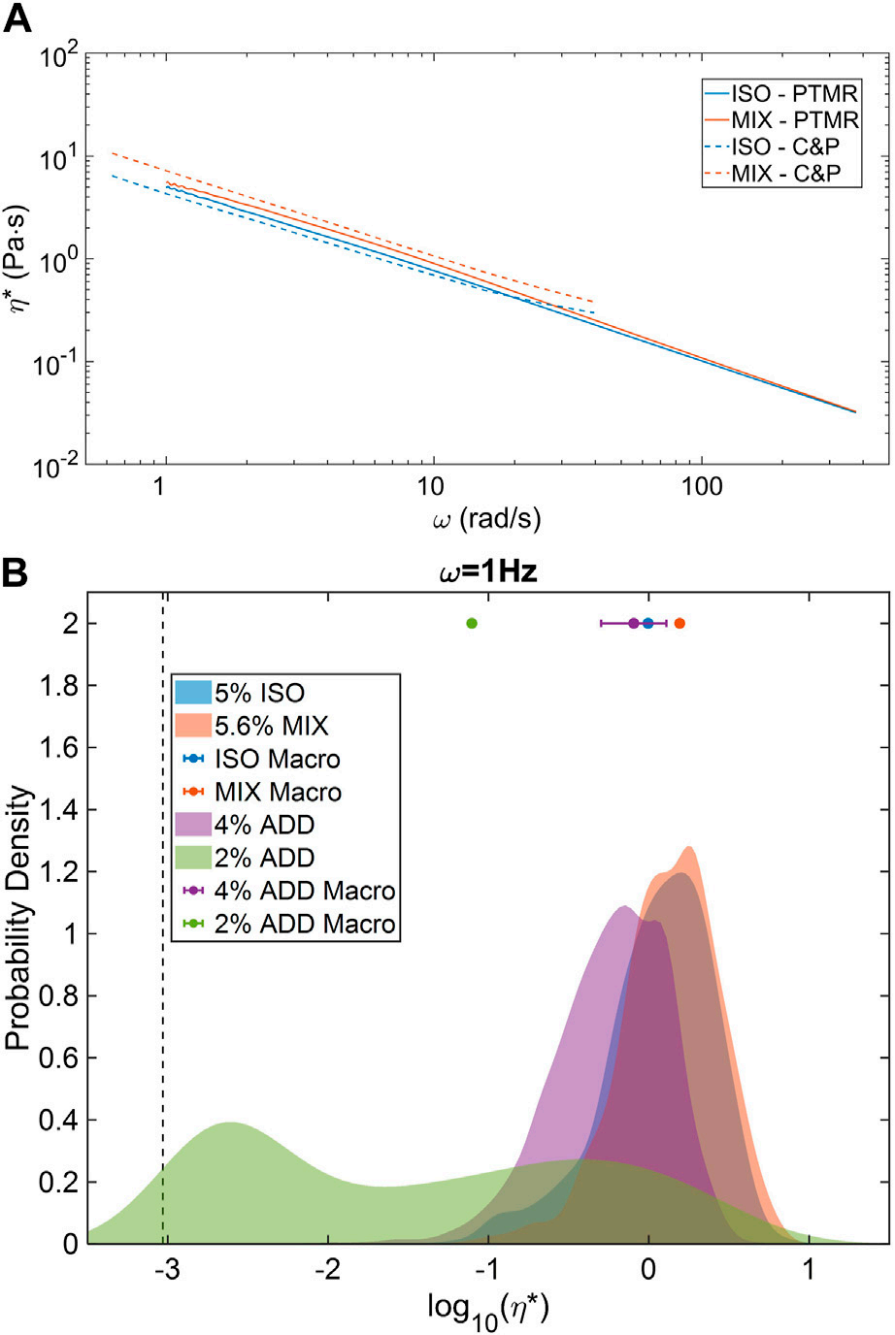
Macro and microrheological comparison of ETT mucus rheology. **(A)** ISO and MIX pools demonstrated strong agreement in complex viscosity across nearly two decades of frequency response. **(B)** Higher % solids samples displayed homogeneous PTMR distributions while 2% ETT displayed a bimodal, low and high-viscosity behavior. However, central tendencies of all distributions were in agreement with mean bulk rheological behavior. Data obtained from *n* = 3 technical replicates.

General agreement between measured bulk values η* and the central tendency of PTMR η* distributions has been previously reported in ETT mucus (Markovetz et al., 2019). Similar agreement was again found between macro and micro scales in both the ISO and MIX pools (Figure 4A). Average frequency-dependent η* was nearly identical between macro and microrheological measurements within each sample type. Concentration-based effects were also apparent at all frequencies. Similar cross-scale agreement was found in both ADD pool concentrations at 1 Hz, and though the PTMR distribution was characteristically bimodal at 2% solids concentration (Figure 4B), the median η* from PTMR was within a factor of 2 of the bulk measurement. These results indicate that airway mucus in the normal to pathological range of concentration has a general biophysical response that can be accurately measured *via* either bulk or microrheological means. However, while bulk measures may be more rapid or accessible, PTMR measurements are able to reveal the complex rheological heterogeneity of normal mucus, particularly nearer to the overlap-entanglement transition where mucus biomacromolecules like mucins begin to interpenetrate, dramatically changing mucus biophysical response (Georgiades et al., 2014).

## 4 Discussion

ETT mucus is a readily available source of human airway mucus that has also been shown to have biophysical and biochemical properties similar to other mucus types used in research like cell culture mucus and induced sputum. Despite its availability and utility, it has yet to see similar usage as other model mucus types. The variability of ETT mucus tonicity may be seen as a limitation since salt content has a definite effect on mucus biophysics (Wagner et al., 2017). However, we have now demonstrated that this can be overcome through characterization of mucus cation content and normalizing tonicity by either mixing or directly modulating salt concentration in pooled samples. The resulting isotonic mucus pools behave rheologically similarly to previously reported ETT mucus samples regardless of the original tonicity of their constituent samples, indicating that most recovered ETT samples can be utilized for research purposes in the absence of blood or other obvious contamination.

The pooled ETT samples studied in this work also serve to reinforce previous reports about the inherent rheological properties of airway mucus at physiological and pathological concentrations. Due to mucus accumulation at the tip of endotracheal tubes during surgery, most samples recovered are hyperconcentrated (∼5% solids), which is roughly equivalent to the concentration of airway mucus in diseases like CF and COPD (Hill et al., 2014). Sample volumes can be somewhat variable but are ∼0.5 mL on average, which is roughly equivalent to the total amount of mucus that can be harvested from cell cultures in our lab on a weekly basis. Given the large volumes of these collections, the ability to maximize the usable number samples presents an opportunity to improve accessibility for laboratory research.

Considering previous results in isotonic ETT mucus, a primary goal of this work was to compare the rheological behavior of a pool of non-isotonic ETT mucus—mixed to be an isotonic pool—to that of a pool of isotonic samples, allowing for greater collection yields and further understanding of mucus behavior in physiological ionic conditions. This was accomplished by pooling an optimal mixture of hypo and hypertonic samples and separately pooling several hypotonic samples and bringing them to isotonicity through NaCl and KCl addition. Both pools behaved as expected, appearing as hydrogels and following established power-law scaling relationships under entanglement proportional to c^4^ at the micro and macro scale. Furthermore, bimodal/biphasic behavior previously observed in samples near the entanglement-overlap transition was recapitulated, indicating that one fraction of mucus is more readily soluble than the other. This may have bearing both in terms of mucus flakes described in BALF (Esther et al., 2019; Markovetz et al., 2022) and also in the concentration-dependent adherence of mucus to airway epithelia that has been recently described (Morrison et al., 2022). Overall, this study furthers the utility and accessibility of ETT mucus as a benchtop model of mucus biophysics and biochemistry, demonstrating that it is a reliably behaved and tunable sample type when attention is paid to maintaining physiological ionic conditions.

## Data Availability

The raw data supporting the conclusion of this article will be made available by the authors, without undue reservation.
